# Hairpins under tension: RNA versus DNA

**DOI:** 10.1093/nar/gkv860

**Published:** 2015-08-31

**Authors:** Mathilde Bercy, Ulrich Bockelmann

**Affiliations:** Laboratoire de Nanobiophysique, ESPCI ParisTech, 10 rue Vauquelin, Paris 75005, France

## Abstract

We use optical tweezers to control the folding and unfolding of individual DNA and RNA hairpins by force. Four hairpin molecules are studied in comparison: two DNA and two RNA ones. We observe that the conformational dynamics is slower for the RNA hairpins than for their DNA counterparts. Our results indicate that structures made of RNA are dynamically more stable. This difference might contribute to the fact that DNA and RNA play fundamentally different biological roles in spite of chemical similarity.

## INTRODUCTION

RNA molecules assemble into a large variety of structures, ranging from a simple hairpin to huge cellular machinery like the ribosome. The structural versatility of RNA is of critical importance to many of the complex functions of RNA, like enzymatic activity, sensing of metabolites and regulated processes, such as transcription termination and attenuation. RNA hairpins participate in the regulation of messenger RNA translation and degradation (1,2). This is made possible by the ability of a hairpin to switch between two distinct conformations, folded and unfolded, where one conformation allows a process and the other one inhibits it. Single stranded DNA can adopt similar secondary structure as RNA and has even been reported to act like an enzyme (deoxyribozyme) *in vitro* (3). It may thus appear surprising that DNA is almost exclusively found in double-stranded form in the biological cell, being only transiently unfolded as for instance during replication or transcription. As an exception single-stranded DNA occurs in certain viruses, but still mainly serves as a hereditary support (4). RNA occupies the domain of complex three-dimensional nucleic acid structures.

With one Watson–Crick paired double helix stem and one single-stranded loop, the hairpin is the simplest secondary structure element and a building block for more complex structures (1). The double helix structures of RNA and DNA are different. RNA double strands adopt an A-form helix that is known to be wider and shorter than the B-form DNA helix (11 nucleotides per turn in the A helix versus 10 in the B helix). RNA and DNA hairpins have been studied separately since the seventies. The first experiments were done with bulk calorimetric techniques (5). Single molecule fluorescence (6) and force (7,8) measurements more recently provided further insight into the kinetics and thermodynamics of hairpins. To the best of our knowledge, no systematic comparison of the folding dynamics of RNA and DNA hairpins has been published so far.

We performed single molecule force measurements with a dual-beam optical trapping setup on a series of molecular constructs containing a DNA or RNA hairpin. Pulling in a direction transverse to the stem, we can impose a mechanical constraint and follow the folding and unfolding of individual hairpins in time. In the main series of experiments, dynamic stretch and release cycles are applied. We used different pulling speeds (corresponding to force loading rates in the range 1-25 pN/s) and extracted kinetic and thermodynamic parameters of the hairpins by comparing the experimental data with a theoretical description based on non-equilibrium statistical physics. Analysing these results, it is crucial to keep in mind the distinction between the activation energy barrier and the equilibrium free energy difference between the two hairpin states (resp. *E* and Δ*G* in the following). There is no general relationship between these two quantities. In addition, the attempt frequency and the force loading rate are the key parameters in this case of non-equilibrium conditions.

We find that the RNA hairpins unfold at higher average force than the DNA hairpins. The force hysteresis, i.e. the difference between transition forces measured during stretching and releasing, increases with pulling speed for all hairpins, in accordance with the theoretical predictions. The RNA hairpins exhibit a much larger force hysteresis than their DNA counterparts. Characteristic transition rates, fitted to the DNA hairpin data, exceed the corresponding RNA rates. This quantitative difference is confirmed and further illustrated by experiments with immobile traps, where the constructs are maintained at constant extension. In this configuration, we find that a large-loop DNA hairpin flips with sub-second dwell-times between its folded and unfolded states, while such flipping was not observable in the corresponding RNA case. As described in the discussion section, our results suggest that RNA structures are less perturbed by mechanical load than DNA structures. The latter tend to switch more rapidly between alternative conformations under the same experimental condition.

## MATERIALS AND METHODS

### Dual-beam optical trapping interferometer

Our dual-beam optical trapping setup relies on a CW Nd:YVO_4_ laser, emitting 2W at 1064 nm (Millenia IR, Spectra Physics, Irvine, CA). The laser beam is split in two beams of orthogonal polarisation. One of the two beams encounters only immobile optical compounds, while the second one is reflected by a mirror mounted on a piezoelectric stage. To avoid issues caused by residual interference between the polarized beams, the mobile beam is frequency shifted with an acousto-optic modulator (9). The two beams are recombined and focused by a 100× oil immersion microscope objective (NA 1.4, Nikon, Tokyo, Japan). This generates two optical traps: a fixed and a mobile one. The setup allows to adjust the distance between the two traps over a range of several micrometers with nanometric precision.

Behind the sample, the beam is collected by a 60× water immersion microscope objective (NA 1.2, Olympus, Tokyo, Japan). The polarisation corresponding to the mobile trap is rejected with a Glan polariser, and the displacement of a bead in the fixed trap is measured by back focal plane interferometry using a PSD detector (Pacific Silicon, Westlake Village, CA, USA). After calibration by analysis of the power spectral density of a captured bead, force is obtained with piconewton precision. More details on the setup have been presented elsewhere (9–11).

### Hairpin molecular constructs

**Figure 1.**
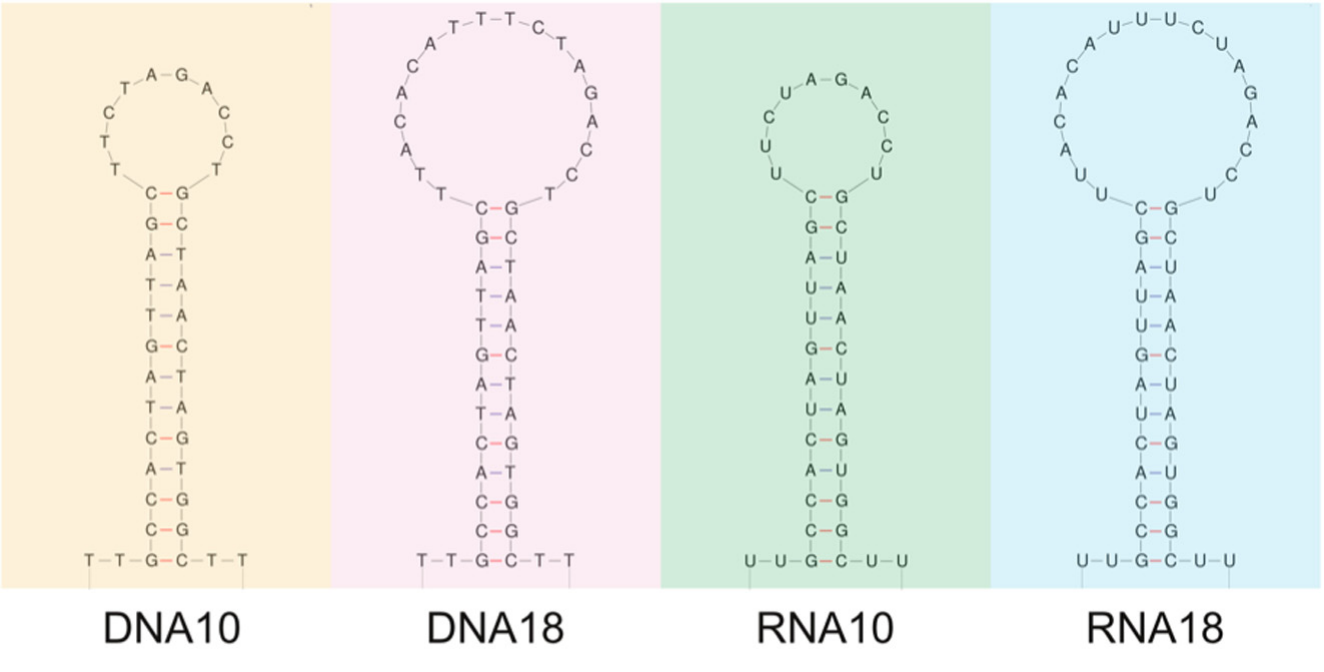
The four studied hairpins. From left to right: DNA10, DNA18, RNA10 and RNA18. They exhibit a 13 base pair stem of the same sequence and loops of either 10 (DNA10 and RNA10) or 18 (DNA18 and RNA18) nucleotides. Apart from the replacement of DNA's Thymine by RNA's Uracil, the base sequences are the same for the DNA and RNA hairpins. The hairpins are presented with their 5′ ends at the bottom left side.

We designed two hairpins with the same stem of 13 base pairs and a loop of 10 or 18 nucleotides, both as an RNA and a DNA molecule. The GC-content of the stem is close to 50 %. The resulting four hairpins are depicted in Figure 1. The colour code used here to distinguish the four hairpins is conserved throughout the article. A distribution of hairpin loop length was determined from the atomic resolution RNA structures available in the Protein Data Bank in January 2013 and it was found that loop sizes between 4 and 20 nucleotides are most frequent (12).

DNA oligonucleotides carrying the hairpin sequence of 36 or 44 bases have been inserted between two double-stranded handles of about 2000 base pairs each. The original DNA corresponds to a sequence coding for 23S ribosomal RNA in an *Escherichia coli* plasmid, which serves as a template for two polymerase chain reactions (PCRs). In addition to the part complementary to the template, one primer of each PCR includes one half of the sequence encoding the hairpin. The PCR products are then digested with Xba1, which generates cohesive ends in the middle of the hairpin sequence. After a ligation step, the complete hairpin sequence appears in the DNA. The resulting ≈4000 nucleotide double-stranded DNA is amplified by PCR. For the RNA hairpin constructs, an *in vitro* transcription of this DNA is performed.

To generate the handles, the DNA strands complementary to the sequence before and after the hairpin part are synthesized by PCR. A digoxigenin functionalized nucleotide is inserted at the 5′ end of one of the handles through the PCR primer. The other handle undergoes an enzymatic treatment (XhoI and Klenow, New England Biolabs, Ipswich, MA, USA) after PCR to add a biotin functionalized nucleotide at the 3′ end (10).

**Figure 2.**
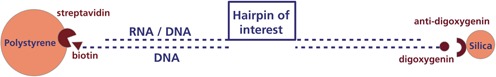
Schematic representation of a molecular construct linked to functionalized beads. A DNA (resp. RNA) hairpin is inserted between two DNA/DNA (resp. RNA/DNA) handles. Each handle has a crystallographic length of about 0.5 μm (resp. 0.4 μm).

To hybridise the handles with the hairpin containing strand, each double-stranded handle and the complete double-stranded DNA (resp. single-stranded RNA) are incubated for 30 s at 95°C, and the sample is cooled down to 4°C over 2 h. Carboxylated polystyrene (resp. silica) microspheres (Bangs Laboratories, Fishers, IN, USA) of 2.19 μm diameter (resp. 1.01 μm) are functionalized (PolyLink Protein Coupling Kit, Polysciences, Warrington, PA, USA) with biotin (resp. anti-digoxigenin) from Roche (Basel, Switzerland). Prior to each experiment, the beads are linked to the molecular constructs by incubation at room temperature (11) (Figure 2).

### Single molecule force measurements

After incubating the molecular constructs with the beads, the sample is diluted in the working buffer (20 mM Tris, 50 mM K acetate, 5 mM Mg acetate, pH7.5) and put in a ≈100 μm deep chamber between two microscope cover slips (11). The chamber is placed between the microscope objectives. The temperature inside the sample during an experiment has been measured. It is 29°C. The experimenter spots a pair of beads linked by a molecular construct, and captures one bead in each trap. The mobile trap displacement is monitored by a custom software programmed in the Labview environment (National Instrument, Austin, TX, USA). After an appropriate anti-aliasing filter, the voltage that is proportional to the force is acquired and displayed in real-time.

### Theoretical description and data analysis

Hairpin folding and unfolding are complex dynamical processes. They depend on several characteristics of the molecular construct. The base sequence of the hairpin stem induces a complex energy landscape and the size of the loop and the elasticity of the DNA/RNA handles also are important. Moreover, the force measurement by itself influences the experimental result, via the trap stiffness and the externally imposed distance versus time protocol. The imposed distance between the centres of the optical traps is subject to position noise arising from piezo-control electronics, mechanical vibration and acoustic perturbations.

**Figure 3.**
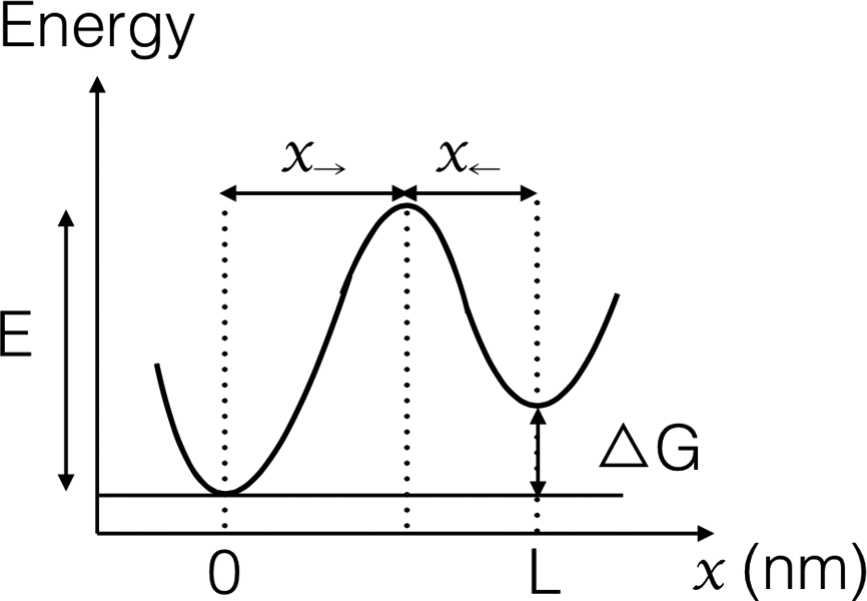
Schematic representation of the simplified energy landscape used in our theoretical description. The state of the folded hairpin occurs at *x* = 0 and the state of the unfolded hairpin at *x* = *L*. The distances between the transition state and the folded and unfolded states are denoted by *x*_→_ and *x*_←_, respectively. By definition, we have *L* = *x*_→_ + *x*_←_.

We use an out-of-equilibrium statistical physics description that is closely related to earlier publications (8,13–17) (usually referred to as Bell-Evans model), and including a minimal set of parameters. We replace the complex molecular energy landscape by the single-barrier landscape presented in Figure 3 and the elasticities of the molecular construct and optical traps are lumped together in one effective stiffness *k*_*eff*_. A free energy barrier of height *E* separates the folded (*x* = 0) and unfolded (*x* = *L*) states. The free energies of the two states differ by Δ*G*. In the absence of force, the folded to unfolded transition rate is *k*_0_ = *k*(*F* = 0) = ν_0_ exp ( − *E*/*k*_*B*_*T*) (it includes the barrier height E and the attempt frequency ν_0_). The reverse process is associated to a transition rate *k*_1_ = *k*_0_exp (Δ*G*/*k*_*B*_*T*) = ν_0_ exp ( − (*E* − Δ*G*)/*k*_*B*_*T*). Applying an external force *F* tilts this landscape. In the limit of high barrier (*E* ≫ *k*_*B*_*T*) and small applied force (*F* ≪ *E*/*x*_→_), the position of the transition state *x*_→_ does not vary significantly and the barrier for unfolding is reduced by an amount *F* *x*_→_. Increasing (resp. decreasing) the external force linearly with time within this model, the unfolding probability *p*_→_(*F*) (resp. folding probability *p*_←_(*F*)) can be derived analytically. More details about the model and a complete derivation of these probabilities are presented in Supplementary Section S1. Both probabilities explicitly depend on the pulling speed.

The distribution of the force hysteresis between unfolding and folding corresponds to the convolution of these probabilities. To obtain the experimental hysteresis distributions, for each molecule the unfolding and folding forces measured are paired to generate all possible hysteresis values (*F*_*unfold*_ − *F*_*fold*_). The obtained values are weighted according to the number of force cycles actually performed on the molecule, and normalized. More details about the building of the histograms are presented in Supplementary Section S3. We numerically calculate *p*_→_(*F*), *p*_←_(*F*) and their convolution, compare the theoretical result with the measured hysteresis distribution and fit the position of the transition state *x*_→_ and a characteristic transition rate *k*_0_. The fit is globally performed on the data of the four velocities in order to obtain a single set of parameters for each hairpin (see Supplementary Section S1). We analyse hysteresis values rather than separate unfolding and folding forces because the uncertainties of the hysteresis values are smaller in our measurements. This is explained by a simple estimation of the experimental errors, presented in Supplementary Section S2. All other parameters are measured, taken from the literature or estimated with the structure prediction program mfold (18). More details about the theoretical description, the parameters determination and the fitting procedure are provided in the Supplementary Sections S1–S3.

## RESULTS

In the beginning of a measurement with varying extension, the mobile trap is moved apart from the fixed trap with a constant velocity. This movement is stopped at a relative extension of the construct (ratio of total length under tension to crystallographic length) of ≈1.4, where the hairpin is always in the unfolded state. The movement is then inversed, so that the traps reapproach at the same speed and the hairpin can refold. This cycle is performed several times on the same molecule.

**Figure 4.**
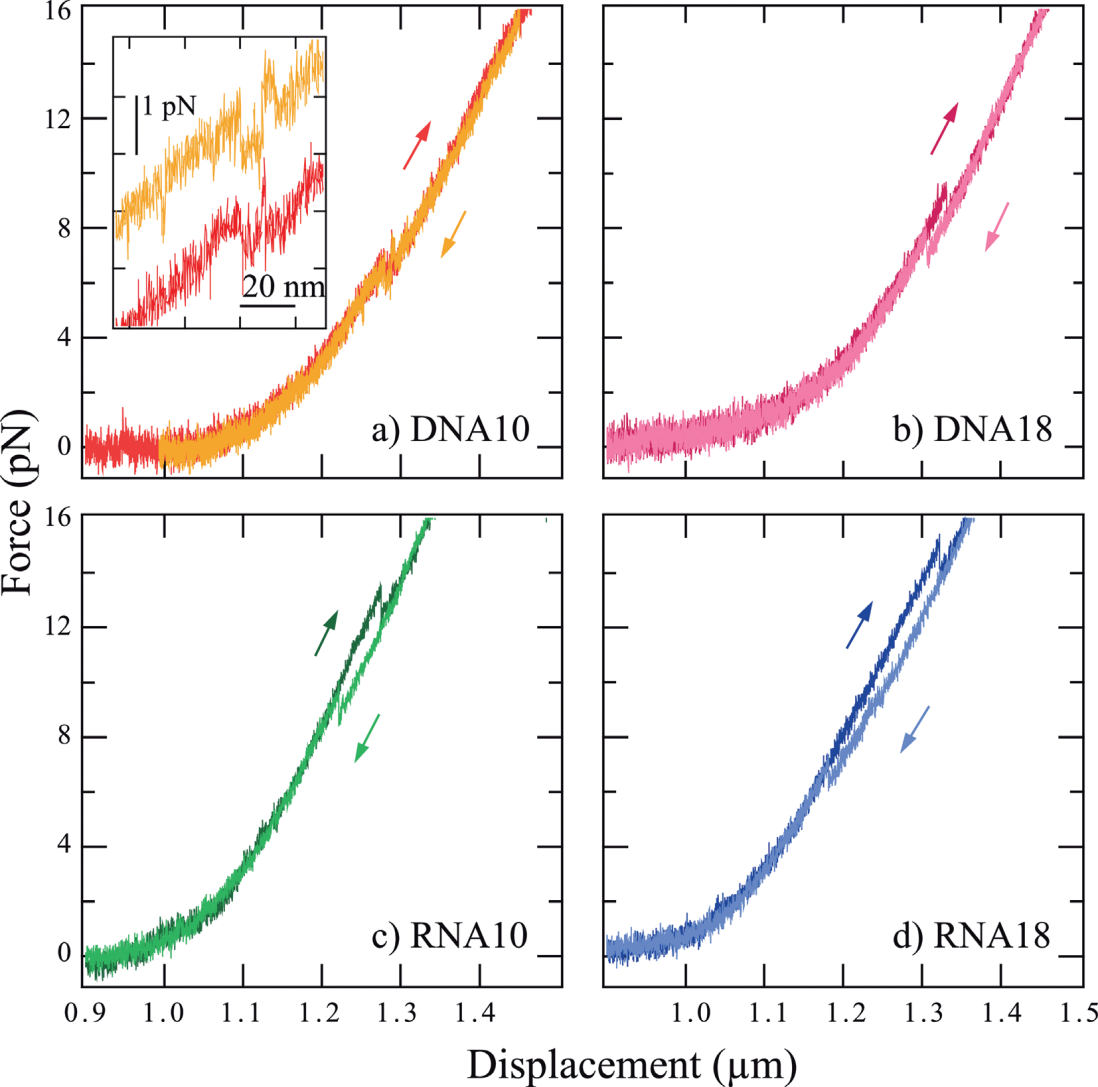
Force versus extension curves at 50 nm/s for the four different hairpins. The darker curves correspond to the stretching of the construct, whereas the lighter ones represent the release process. For both the DNA and RNA hairpins, the hysteresis between unfolding and folding increases with loop length. For the same hairpin sequence, the hysteresis is larger for the RNA construct than for the DNA one. The inset provides a closer look to the force flips observed with DNA10. In this inset, the unfolding and folding curves are shifted vertically for clarity.

In Figure 4, we present typical force versus extension curves for the four hairpins at 50 nm/s. The left part of each curve (small displacement range not entirely shown) corresponds to the entropic response of the double-stranded handles where the force remains small. Force increases with a rising slope when the displacement approaches the crystallographic length of the molecular construct. This regime corresponds to the elastic response of the double-stranded handles, well described by the Worm Like Chain model (19) (see Supplementary Section S4, Supplementary Figure S1). For the DNA hairpin constructs, the handles are double-stranded DNA, whereas the RNA hairpin constructs have hybrid DNA/RNA handles. This explains the length difference at the starting point of the elastic response. At a certain extension, a sudden drop in force by about 1 pN is observed, which corresponds to the unfolding of the hairpin. After this event, the force increases again, now stretching the whole construct with the double-stranded handles and the unfolded hairpin in series. No intermediate state is observed during hairpin unfolding.

When releasing the strain by inversing the direction of displacement and bringing the traps closer to each other, the hairpin can fold back into its initial structure. As no intermediate state is observed during the corresponding sudden upwards rise in force, this process also appears to be cooperative.

**Figure 5.**
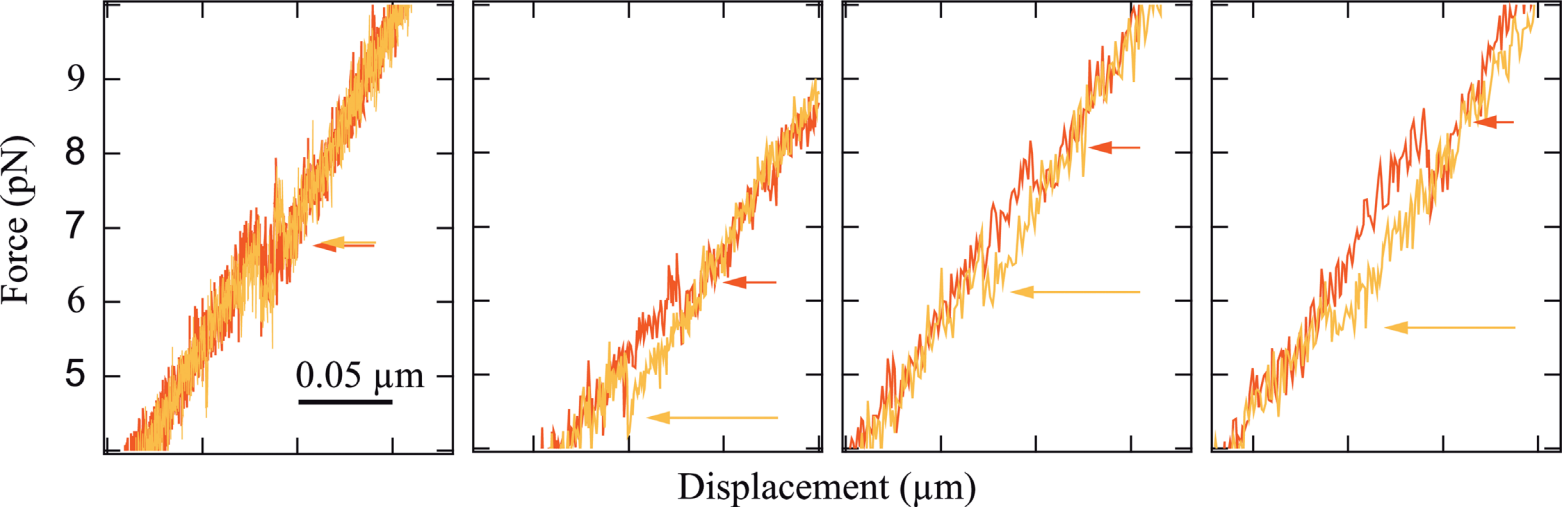
Force versus extension curves for DNA10 at 50, 150, 300 and 450 nm/s (from left to right). The darker curves correspond to the stretching of the construct, whereas the lighter ones represent the release process. The arrows indicate the force of the unfolding and folding events. The force flips observed with DNA10 at 50 nm/s vanishes at 150 nm/s and above. The hysteresis between unfolding and folding increases with the pulling velocity.

The stretch and release curves are indistinguishable, except for the region where the unfolding (resp. folding) occurs. For a velocity of 50 nm/s and for all constructs except DNA10, a force hysteresis between unfolding and folding is observed. The hysteresis indicates that transitions occur out of thermal equilibrium. On the other hand, DNA10 shows several force flips between the folded and unfolded states during dynamic stretching and releasing of the construct (Figure 4a, inset). These force flips are a signature of a close-to-equilibrium dynamics (20). They are no longer observed and hysteresis becomes notable when the displacement velocity is increased to 150 nm/s and above (Figure 5). No force flips are observed for the other three hairpins.

Experimental data presented in Table 1 show that, both for the DNA and RNA hairpins, the most probable folding force decreases with increasing loop size, while the unfolding force is less sensitive to this parameter. Both the unfolding and the folding forces of the RNA hairpins are higher than the corresponding forces of the DNA hairpins.

**Table 1.** Most probable folding and unfolding forces, determined from measurements at a velocity of 50 nm/s. See Supplementary Figure S2 for an example of unfolding and folding forces distribution

Hairpin	Unfolding force	Folding force
	(pN)	(pN)
DNA10	6.4	6.2
DNA18	6.1	3.6
RNA10	13.6	8.8
RNA18	14.1	5.8

The presented force values correspond to the maxima of a Gaussian fit of the measured distributions of the forces where unfolding or folding occurs. The unfolding force distributions are build from 77 measured unfolding events for DNA10, 93 events for DNA18, 179 events for RNA10 and 146 events for RNA18. The folding force distributions are based on 79 measured folding events for DNA10, 70 events for DNA18, 148 events for RNA10 and 114 events for RNA18. The widths of these distributions are about 2.5 pN (FWHM).

**Figure 6.**
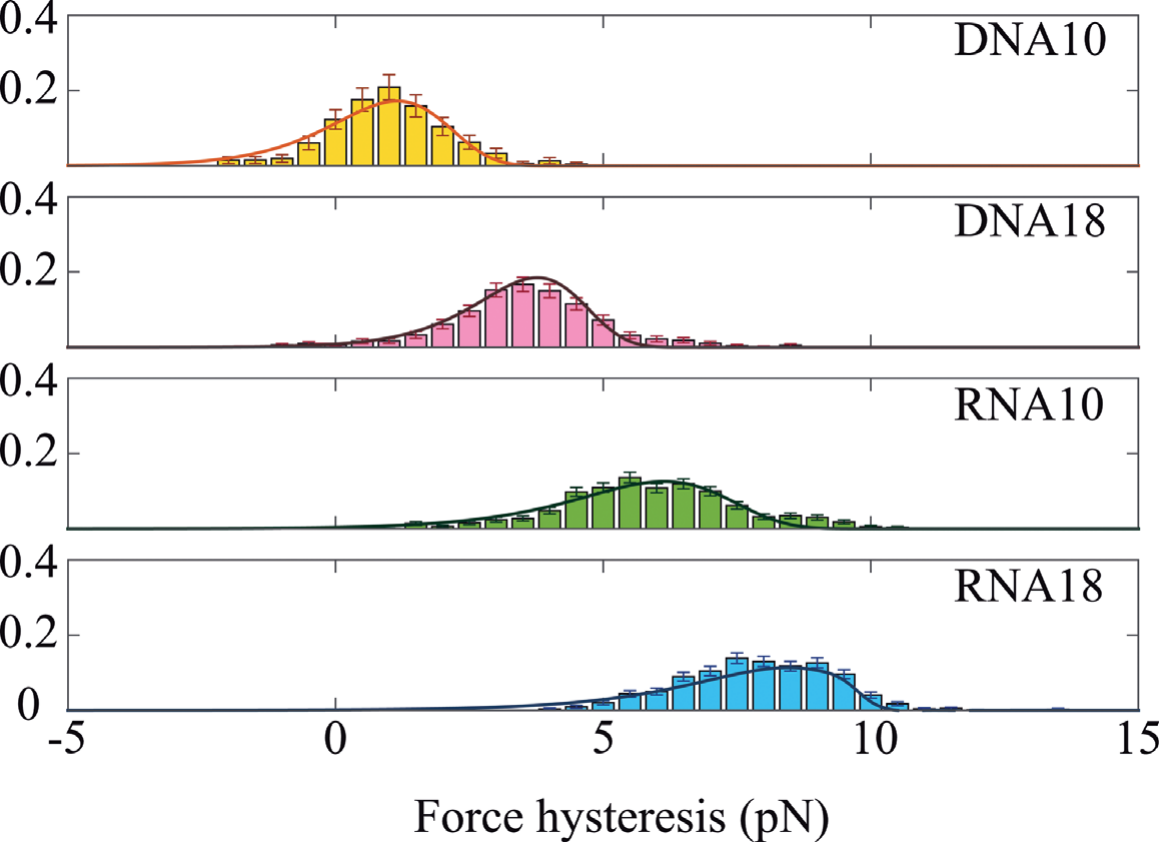
Histograms of the hysteresis measured at 150 nm/s on the four different hairpins. The experimental distributions are fitted to calculated convolution of the unfolding and folding probability distributions (solid line), as explained in Materials and Methods and Supplementary Section S1 (Equations (4–6)). For each fitted histogram, the root mean squared error (*rmse*) is in the range (2.0–3.0) × 10^−3^. For DNA10 the data come from 46 stretch/release cycles on 12 molecules. DNA18: 95 cycles on 13 molecules. RNA10: 152 cycles on 44 molecules. RNA18: 150 cycles on 21 molecules.

Measuring the successive unfolding (resp. folding) forces during stretch/release cycles on different molecules allowed us to build the hysteresis histograms for the four hairpins at the different displacement velocities. For DNA10 at 50 nm/s, when flips occur, only the first unfolding and first refolding forces are measured, according to the model used. Figure 6 shows such histograms for the four hairpins at 150 nm/s. The histograms corresponding to the other velocities are presented in Supplementary Section S6 and Supplementary Figure S3. Our data show that for a given hairpin structure and under identical experimental conditions (same experimental buffer, same temperature, same pulling speed), the hysteresis is notably higher for RNA than for DNA. We find that the hysteresis increases with loop length for both DNA and RNA.

For all four hairpins, the force hysteresis increases with displacement velocity. Supplementary Figure S4 (Supplementary Section S7) presents histograms of the hysteresis recorded for RNA10 at 50, 150, 300 and 450 nm/s as an example. A clear increase of the mean hysteresis is visible, with its value going from 5 pN at 50 nm/s to 7.5 pN at 450 nm/s. The hysteresis values for the four hairpins are given in Supplementary Table S2. For each hairpin and pulling speed, variability in the unfolding and folding forces is seen, inducing a variability in the hysteresis. This behaviour is expected from the stochastic character of the unfolding and folding processes.

Theoretical analysis of the hysteresis histograms enables us to extract thermodynamic and kinetic information about the hairpin structures. The experimental distributions were fitted to calculated probability distributions, as defined in Materials and Methods and supplementary information. For each hairpin, the fitting procedure is applied over the global data set, combining the four different speeds. The calculated force hysteresis distribution is presented by the solid line in Figure 6.

Measured mean transition forces *F*_*t*_ (average of the mean opening force and the mean folding force at 50 nm/s) and transition lengths *L*, as well as theoretical free energy values that were obtained by simulation (see Supplementary Section S1 for details) are presented in Table 2. Two parameters, the distance *x*_→_ between folded state and transition state and the characteristic transition rate *k*_0_ were fitted to the experimental data and are presented in Table 3. The quality of the fits is good, with a root mean squared error in the range (1.0–5.0) × 10^−3^ for each fitted histogram (*rmse*, see Supplementary Section S1, Equation (7) for definition) and allows us to univocally determine the two parameters.

**Table 2.** Data obtained from measurements at 50 nm/s (a) or theoretical prediction (b)

	(a)	(a)	(b)	(b)	(b)
Hairpin	Transition	Transition	Δ*G*	Δ*G*	Δ*G*
	force *F*_*t*_	length *L*	mfold	stretch	total
	(pN)	(nm)	(kJ/mol)	(kJ/mol)	(kJ/mol)
DNA10	6.3	14.8 ± 2	63± 3	20 ± 1	83 ± 4
DNA18	4.85	16.6 ± 2	60 ± 3	18 ± 1	78 ± 4
RNA10	11.2	17.7 ± 2	89 ± 4	32 ± 2	121 ± 6
RNA18	9.95	18.4 ± 2	87 ± 4	35 ± 2	122 ± 6

mfold: http://mfold.rna.albany.edu, see Materials and Methods and Supplementary Information for parameter determination.

**Table 3.** Parameters deduced from hysteresis measurements

Hairpin	Transition state	Transition state	Transition rate
	*x*_→_	*x*_→_/*L*	*k*_0_
	(nm)		(*s*^−1^)
DNA10	4.4 ± 0.2	0.30	(1.0–3.0) × 10^−4^
DNA18	4.7 ± 0.2	0.28	(3.8–8.5) × 10^−5^
RNA10	3.0 ± 0.2	0.17	(2.9–5.5) × 10^−5^
RNA18	3.1 ± 0.2	0.17	(3.0–10) × 10^−6^

By definition, we have *x*_←_ = *L* − *x*_→_ and *x*_←_/*L* = 1 − *x*_→_/*L*, *k*_0_ = *k*(*F* = 0) = ν_0_ exp ( − *E*/*k*_*B*_*T*)

The results for *x*_→_ indicate a clear asymmetry in the energy landscape for both DNA and RNA hairpins (all ratio *x*_→_/*L* < 0.5). The DNA values of *x*_→_/*L* ≈ 0.3 are higher than the RNA values of *x*_→_/*L* ≈ 0.17. The transition rates *k*_0_ differ by a factor of about 10 between DNA18 and RNA18. The ratio is also about 10 between DNA10 and RNA10. These results confirm and quantify the faster dynamics of DNA as compared to RNA, already suggested by the appearance of force flips in the DNA10 curves at 50 nm/s.

The observation that the transition rates and hence the dynamics differ between the DNA and RNA hairpins is further confirmed by additional measurements performed with the four hairpins at constant extension. Here, the molecular construct is brought to a defined extension, which is then maintained constant during 10 s to 2 min. If the energy barrier is not too high, there are extension values where the hairpin can spontaneously flip between its folded and unfolded states (7,20); the measured force flips are induced by thermal fluctuations. If the corresponding dwell times do not significantly exceed the time-scale of the measurement, these spontaneous transitions can be observed experimentally.

**Figure 7.**
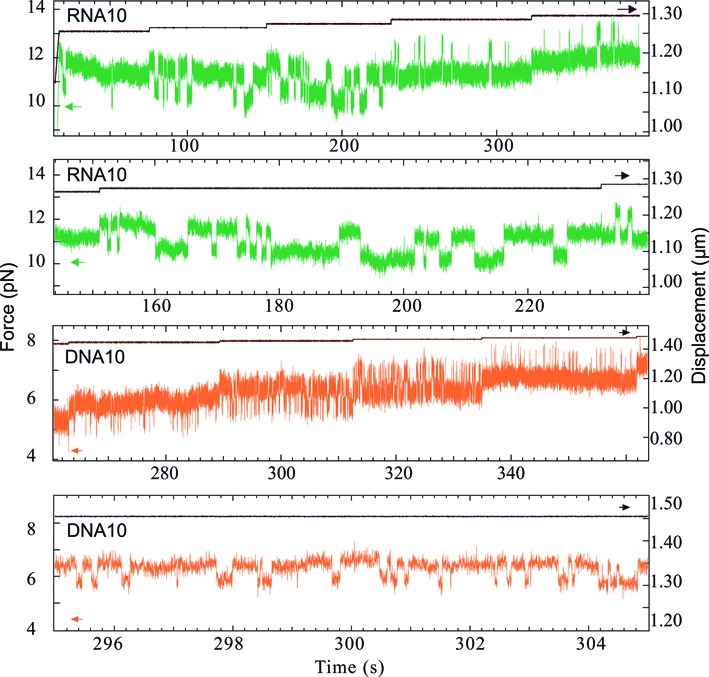
Spontaneous transitions between folded and unfolded hairpin states at constant extension. (Top panel) Force recorded on RNA10 (green) as a function of time, while the extension is constant apart from stepwise increases (black). Force flipping is observed for each extension value. (Second panel) Zoom on an extension where both states have a similar probability of occupation: the average dwell times are 1.77 s in the unfolded state and 3.1 s in the folded state. Presented time range: 80 s. (Third panel) Force recorded on DNA10 (orange) as a function of time, while the extension is constant apart from stepwise increases (black). Force flipping is observed for each extension value. (Bottom panel) Zoom on an extension where both states have a similar probability of occupation: the average dwell times are 0.11 s in the unfolded state and 0.25 s in the folded state. Presented time range: 10 s. The transition rate is much higher for DNA10 than for RNA10.

Figure 7 (top) shows the force recorded on RNA10 as a function of time, while extension is held constant apart from stepwise increases. At the smallest extension, the hairpin remains folded most of the time, but already displays some brief passages to the unfolded state. The mean lifetime in the unfolded state progressively increases with extension, and at a given point equals the one in the folded state (this situation is presented in the second panel). At even larger extensions, the unfolded state becomes energetically favorable, and the hairpin only folds sporadically. The same global behaviour is observed for DNA10 (two bottom panels), but the transitions occur more frequently and the dwell-times of the folded and unfolded states are shorter than for RNA10. This observation is in line with the different *k*_0_ values of RNA10 and DNA10, presented in Table 3.

DNA10, DNA18 and RNA10 present spontaneous force flips at constant extension. For RNA18 however, no extension was observed where both spontaneous unfolding and folding occurs, although the two RNA hairpins are close in terms of the theoretically predicted Δ*G* values. This difference between RNA10 and RNA18 is consistent with the observation that the *k*_0_ value of RNA18 obtained from the varying extension data is an order of magnitude smaller than the one of RNA10.

## DISCUSSION

We observe that the RNA hairpins unfold and fold at higher force than the DNA hairpins. This may be expected from the known fact that in terms of free energy difference Δ*G* an RNA duplex is more stable than the DNA duplex of equivalent sequence. Note however that there are three problems with this simplified view. First, the denaturation mechanisms under force and by thermal melting are different (21). Whereas thermal melting involves independent and stochastic fluctuations of the stem bonds until denaturation occurs, force-induced unfolding is a directed process. Even if the bonds still fluctuate, the stem is sequentially opened from the bottom of the hairpin to the top. Similarly, the initial conditions for hairpin thermal folding are different from the ones for force reduction. Whereas folding of a thermally denatured molecule starts from a relatively compact conformation, the force measurement imposes a fully stretched initial construct. Second, the unfolding force can be estimated by a relation of the type *F* = Δ*G*/*L* only if the transition occurs sufficiently close to thermal equilibrium. In the non-equilibrium case the actual barrier height, the attempt frequency and the force loading rate are the main parameters. There is no general relation between the equilibrium Δ*G* and the barrier height. Third, the elastic energy usually plays a significant role in force-induced unfolding. If for the sake of simplicity we consider the equilibrium case, the estimation of the opening force is of the form *F* = Δ*G*_*tot*_/*L*, but the molecular elasticity influences both the characteristic energy (Δ*G*_*tot*_ = Δ*G*_*mfold*_ + Δ*G*_*stretch*_, in the notations of Table 2) and the characteristic length *L* (*L* is the sum of the molecule length at zero-force and the force-induced extension). As can be seen from Table 2, the stretching energy contributions are sizeable for both DNA and RNA.

The hysteresis is larger for RNA18 than for RNA10 and the same holds for DNA18 compared to DNA10. This mainly arises from a loop-length dependence of the folding force. As shown in Table 1, the folding forces significantly decrease with increasing loop size, while the unfolding forces change weakly. To initiate hairpin folding a loop must form to approach the bases of the stem for base pairing. As folding occurs against an external force *F* in the present case, this initiation requires a mechanical work of the order *W* = *FL*, where *L* denotes the length of the unfolded loop. For similar *W* (energy fluctuations of similar amplitude), the different *L* thus implies that folding of the small-loop hairpin occurs at higher *F* than folding of the large-loop one. Of note, it was found by fluorescence techniques that even without external force the rate of hairpin closing decreases with increasing loop size (6).

We find that the transition rate *k*_0_ is smaller for the RNA hairpins than for their DNA counterparts. As shown in Figure 7, the RNA hairpin that exhibits smaller *k*_0_ also shows longer dwell times in both hairpin states. Smaller *k*_0_ leads to larger hysteresis in the constant velocity measurement, since both unfolding and folding happen statistically later on the force ramp. This relation between *k*_0_ on the one hand and magnitude of the force hysteresis on the other hand is observed in our measurements. RNA10 (resp. 18) exhibits smaller *k*_0_ and larger hysteresis than DNA 10 (resp. 18) at given velocity. For example, the transition length *L* corresponding to RNA10 unfolding (resp. folding) is ≈20 nm. Considering a typical equilibrium dwell time of 1 s for this hairpin (estimated from Figure 7, second panel), a pulling velocity of 50 nm/s induces a displacement of 50 nm during this time. This displacement is larger than *L*, and the folding (resp. unfolding) transition is thus highly unlikely. The unfolding transition (resp. folding) thus occurs out of equilibrium at this speed. On the contrary, the shorter dwell time of DNA10 (≈0.1 s) induces a displacement (≈5 nm) that is smaller than *L* (≈15 nm) and allows for flipping between the two states. In this case, the measurement can be considered as close to equilibrium (Figure 4a, inset).

As shown in Table 3, the transition state is closer to the folded state than to the unfolded state. We convert the values of *x*_→_ to the number of unfolded nucleotides, using a procedure described earlier (27). For DNA10 (resp. DNA18) the transition state corresponds to 9 (resp. 11) unfolded nucleotides, or base pair 5–6 of the hairpin stem counted from the bottom. For RNA10 and RNA18 we obtain 5 (resp.6) nucleotides, corresponding to base pair 3 from the bottom. We tentatively attribute these positions to the stem sequence: the first base pairs from the bottom are three G·C (Figure 1), conferring a greater stability to the bottom compared to the top of the stem. This interpretation is consistent with the fact that no intermediate state is observed during hairpin unfolding. Simply speaking, when the mechanical work becomes sufficient to unfold the initial G·C rich regions, the whole stem bursts open cooperatively. Within our model that assumes a single transition state, the folding of the hairpin is correspondingly expected when the G·C rich part of the stem reforms. The fact that no intermediate states were observed in our measurements indicates that the folding and unfolding transitions occur with a high degree of cooperativity. For comparison, we have calculated unfolding free energy landscapes of the hairpins. This study is described in section S8 of the supplementary information. The calculations are based on mfold free energies, include the sequential energy contributions of the different base pairs of the stem and the single-stranded loop, but do not include cooperativity. Interestingly the calculated landscapes exhibit their maxima at the top of the hairpin stem, in contrast to the measurements indicating transition states closer to the bottom of the stem. The differences are significant. First, they provide additional support to the idea that cooperativity is important for all investigated hairpins. Moreover, the comparison suggests a higher degree of cooperativity in RNA than in DNA, since we experimentally observe that the RNA hairpins exhibit smaller *x*_→_ than the DNA hairpins.

There are structural arguments both for the stem and the loop that support a stronger cooperativity of the RNA hairpin as compared to the DNA hairpin. The stem of the RNA hairpin exhibiting a A-form helical structure is shorter and therefore more compact than the stem of the DNA hairpin, which is a B-form double helix. In the RNA helix, the constraint caused by the opening fork may thus more easily extend over several base pairs than in the DNA helix. It is expected that interactions between nucleotides in the hairpin loop contribute in a non-negligible manner to the overall stability. Indeed RNA is known (and DNA suspected) to build non Watson–Crick base pairs and we note that mfold does not fully take the corresponding free-energy contributions into account (22,23). The 2′-OH group of the RNA nucleotide forms hydrogen bonds, while the 2'-H of DNA does not. In this regard, nuclear magnetic resonance studies of chimeric RNA stem/DNA loop hairpins revealed that 2′-H in the hairpin loop reduces the overall stability and also suggests that the structure of the stem, A-form or B-form, strongly influences the loop structure (24).

We tentatively attribute the slower dynamics of the RNA hairpins to a stronger cooperativity. For strong cooperativity, barriers against folding and unfolding may be localized and high, since they can cumulate energy contributions of many structural elements. With weak cooperativity, on the other hand, the energy contributions of the structural elements distribute more equally over the reaction coordinate, high local barriers are less probable to occur and therefore passage of this landscape by a random walk that is biased by the external force is faster.

Measurements on more complex RNA structures showed pronounced structures in the force versus extension curves (or length versus force curves for force-clamp measurements) and hysteresis occurred even at low pulling speed (25–27), in qualitative agreement with the present findings. Recently, an investigation of the mechanical properties of double-stranded RNA under force and torque has been published (28). The authors applied torque to a torsionally constraint double-stranded RNA construct using magnetic tweezers. They investigated in particular a plectonemic buckling transition. They find that the characteristic transition rate of this buckling transition is two orders of magnitude smaller for double-stranded RNA than for double-stranded DNA. It is interesting to mention this study in the present context because it shows a slower dynamics for RNA than for DNA, although the buckling transition is different from hairpin folding.

A typical RNA molecule has a complex three-dimensional shape stabilized by several duplexes. The individual duplexes are connected by single strands of varying length, which allows for propagation of conformational changes within the RNA structure. This propagation of strain and displacement is influenced by the elasticity of the connecting single strands. Forces of the order *k*/Δ*x* develop, where *k* is the stiffness of the molecular linker and Δ*x* the relative displacement. This global picture suggests that the configuration studied in the present paper represents an elementary building block of complex three-dimensional structures formed by a single-stranded nucleic acid chain. Specifically, our result that the dynamics of folding and unfolding under mechanical load is slower in RNA hairpins than in the corresponding DNA hairpins translates itself into predicting that RNA structures are dynamically more stable. In other words, structures formed by single-stranded DNA rather than RNA exhibit significantly faster inter-conversion between alternative configurations. There are of course good reasons of evolutionary origin to explain why the domain of biologically-active three-dimensional nucleic acid structures is traditionally occupied by RNA. Our suggestion that RNA structures are dynamically more stable than DNA structures may be an interesting additional argument to understand why single-stranded DNA was not selected to become a molecule that assures its biological role by a complex internal structure.

## CONCLUSION

Two RNA hairpin structures of different loop size were compared with their DNA equivalents. Under the same experimental conditions, the forces needed to unfold the DNA hairpins are systematically lower. Hysteresis between unfolding and folding is more pronounced for RNA than for DNA. For all studied hairpin types, the hysteresis increases with the pulling speed. Imposing a constant distance, spontaneous force flips are observed for the large loop DNA hairpin, but are absent for the equivalent RNA structure. For small loop hairpins, flipping between the folded and unfolded states occurs with both DNA and RNA. Comparison of the experimental data with a theoretical description allowed us to estimate the position of the transition state and a characteristic transition rate. The latter is found to be smaller for RNA than DNA. From all these results emerges the picture that RNA exhibits a more pronounced out-of-equilibrium character than DNA. The force-induced conformational dynamics of hairpins is slower in RNA than in DNA. The results suggest that structures made of RNA are more stable against internal and external forces than structures made of single-stranded DNA and therefore the former are more apt to form complex structures that are required for an active biological function.

## SUPPLEMENTARY DATA

Supplementary Data are available at NAR Online.

SUPPLEMENTARY DATA
