# Supplementary material for: Hairpins under tension: RNA versus DNA
Source: Nucleic Acids Res. 2015 Aug 31;43(20):9928–36. doi: 10.1093/nar/gkv860 (PMC4787782; doi:10.1093/nar/gkv860)
Supplement: SUPPLEMENTARY DATA [file supp_43_20_9928__index.html]

Hairpins under tension: RNA versus DNA — SUPPLEMENTARY DATA 

# Hairpins under tension: RNA versus DNA

## SUPPLEMENTARY DATA

- SUPPLEMENTARY DATA
