# Supplementary material for: Hairpins under tension: RNA versus DNA
Source: Nucleic Acids Res. 2015 Aug 31;43(20):9928–36. doi: 10.1093/nar/gkv860 (PMC4787782; doi:10.1093/nar/gkv860)
Supplement: SUPPLEMENTARY DATA [file supp_gkv860_nar-01492-f-2015-File002.pdf]

Hairpins under tension: RNA versus DNA
# - Supplementary Information -

Mathilde Bercy and Ulrich Bockelmann

Laboratoire de Nanobiophysique
ESPCI ParisTech, 10 rue Vauquelin, 75005 Paris, France

S.1 Theoretical description and parameters determination

In this supplementary section, we first present analytical expressions for the unfolding and folding probabilities and the force hysteresis. Afterwards, the parameters are presented and the relation between our theoretical description and earlier publications is considered. Then, we describe how each parameter is obtained, by direct measurement, from the literature, by mfold calculations or by data fitting. Finally, we consider the fitting procedure and how its quality is quantified.

Theoretical description

Let us start considering the two-state free energy landscape presented in main text Fig.3. At $t = 0$, the molecule is in the folded state ($x = 0$). Force increases with a constant rate r according to

$$F = r\,t = k_{eff}\,v\,t \tag{1}$$

with an effective stiffness k_{eff} and a displacement velocity v. We define $S(F)$ as the probability that the molecule is still folded at time t when the force has increased to a value F. Assuming first order kinetics, we have

$$\frac{dS}{dt} = -k\left(F\left(t\right)\right)\,S(t) \tag{2}$$

where $k(F)$ is a force-dependent transition rate from the folded to the unfolded state. Assuming $k(F) = \nu_0\,\exp\left(-(E - F\,x_{\rightarrow})/k_BT\right)$

with an attempt frequency ν_0, Eq.2 is easily solved for the linear force ramp of Eq.1. :

$$S(F) = exp\left(-\frac{1}{r}\int_0^F k(F')dF'\right)$$

Introducing the distribution of rupture forces $p(F)$ that is related to $S(F)$ by

$$\frac{dS}{dt}dt = -p(F)dF$$

we obtain the unfolding probability [1]

$$p_{\rightarrow}(F) = \frac{k(F)}{r}exp\left(-\frac{1}{r}\int_0^F k(F')dF'\right) \tag{3}$$

and thus

$$p_{\rightarrow}(F) = C_{\rightarrow}\exp\left(\frac{F\,x_{\rightarrow}}{k_BT} - \frac{k_0\,k_BT}{r\,x_{\rightarrow}}\exp\left(\frac{F\,x_{\rightarrow}}{k_BT}\right)\right) \tag{4}$$

where $k_0 = k(F=0) = \nu_0\,\exp(-E/k_BT)$.

The same procedure can be used for the folding probability $p_{\leftarrow}(F)$, which leads to

$$p_{\leftarrow}(F) = C_{\leftarrow}\exp\left(-\frac{F\,x_{\leftarrow}}{k_BT} - \frac{k_1\,k_BT}{r\,x_{\leftarrow}}\exp\left(-\frac{F\,x_{\leftarrow}}{k_BT}\right)\right) \tag{5}$$

with $k_1 = k_0\exp(\Delta G/k_BT) = \nu_0\,\exp(-(E-\Delta G)/k_BT)$.

$C_{\rightarrow}$ and $C_{\leftarrow}$ in Eqs.4-5 are normalisation factors which comes out when Eq.3 (and the corresponding one for $p_{\leftarrow}$) is calculated. They ensure the normalisation $\int_0^{+\infty} p_{\rightarrow}(F')dF' = 1$ (resp. $p_{\leftarrow}$).

$$C_{\rightarrow} = \frac{k_0}{r}exp\left(\frac{k_0\,k_BT}{r\,x_{\rightarrow}}\right)$$

and

$$C_{\leftarrow} = \frac{k_1}{r}$$

[1] Strictly speaking, $p_{\rightarrow}(F)$ and $p_{\leftarrow}(F)$ are probability densities, of dimension N^{-1}. For the sake of simplicity, we have retain the simple notation "probability". In the figures, we present real probabilities, i.e. the probability density is integrated over the force interval of the bin.

provided that the starting force of the decreasing force ramp is large enough to prevent folding events. In that case, the exact initial force doesn't influence the folding probability. In our experiments, 20 pN ensures this condition for all the hairpins studied.

The sum of two distributions (which is also a distribution) corresponds to their convolution. The distribution of hysteresis thus corresponds to the convolution of the distributions $p_{\rightarrow}(F)$ and $p_{\leftarrow}(-F)$:

$$p_{hys}(\Delta F) = (p_{\rightarrow} * p_{\leftarrow})(\Delta F) = \int_{-\infty}^{+\infty} p_{\rightarrow}(F) p_{\leftarrow}(F - \Delta F) dF \qquad (6)$$

$p_{\rightarrow}(F)$ and $p_{\leftarrow}(-F)$ are calculated numerically using Eqs. 4-5, and their convolution is computed by the corresponding Matlab function.

Parameters and relation to theoretical descriptions of the literature

This model corresponds to the one described in the literature first by Evans (1) (relying on former work by Kramers (2) and Bell (3)) and more recently enriched by Dudko and coworkers among others (4, 5), particularly by taking into account various geometries for the landscape shape. In the aim of using a small number of parameters for our comparison, we choose to consider the case of a linear force ramp and use Bell's formula to describe $k(F)$ (3). This doesn't introduce new parameters for the shape of the landscape. This description involves a set of 3 parameters for the unfolding process, namely $x_{\rightarrow}$, $k_0 = \nu_0 \exp(-E/k_B T)$, and $r = k_{eff} v$. Two additional ones are needed for the folding process: $x_{\leftarrow}$ and k_1. Note that the attempt frequency ν_0 and the energy barrier E are not considered separately, but merge in the effective attempt frequency k_0.
Since L can be measured, we express $x_{\leftarrow}$ as $L - x_{\rightarrow}$. We assume the same attempt frequency ν_0 for unfolding and folding, yielding to $k_1 = k_0 \, exp(\Delta G / k_B T)$. With ΔG obtained by calculation (mfold and stretching correction), k_1 is defined by the knowledge of k_0. In addition, the loading rate r is determined since the effective stiffness k_{eff} is measured, and the velocity v experimentally imposed. The measurement and calculation procedures for these parameters are described in the following section. This leaves us with only two fitting parameters, namely $x_{\rightarrow}$ and k_0.

Parameter determination

The total generated length $L = x_{\rightarrow} + x_{\leftarrow}$ is measured for each hairpin at the mean transition force F_t, which is the average of the mean unfolding force and the mean folding force at 50 nm/s (main text Table 2).

The effective stiffness k_{eff} (needed to calculate the loading rate r, see Eq. 1) is obtained from the local slope of the recorded force versus displacement curve. The slope is measured on the 25 nm preceding the transition. It is measured at each unfolding event, and for each hairpin the average of these measurements is used.

ΔG is calculated for each hairpin. It has two contributions: the free energy difference between the folded and the unfolded hairpin structures at zero-force and the elastic energy required to stretch the unfolded chain from zero-force to the average transition force F_t. The first contribution is estimated using the secondary structure prediction software mfold that gives free energies at 29 °C and 1 M NaCl. As we work at this temperature but with different ionic conditions, free energies had to be corrected for the difference in salinity. Mfold provides such salt correction for DNA, but not for RNA. For an RNA hairpin we thus apply the same correction as for the DNA hairpin of identical sequence (replacing U by T). The stretching energies are calculated by integrating theoretical force versus extension curves. The worm-like chain (WLC) model is assumed for the DNA and RNA single stranded nucleic acid chains. A persistence length l_p of 1.2 nm and a crystallographic length per nucleotide l_0 of 0.70 nm are used for DNA, while for RNA we take $l_p = 1.37$ nm and $l_0 = 0.65$ nm, respectively. These parameters were the best match with our measured L and F_t values (Table 2, main text), and are in good agreement with the ones from the literature (6–9).

Fitting procedure

The theoretical force hysteresis distribution is used as fitting function for the measured force hysteresis distributions, with $x_{\rightarrow}$ and k_0 as fitting parameters. The fits are done with a Matlab least-square fit algorithm. For each hairpin, the hysteresis distributions of the four velocities are fitted together as follows. In a first step, independent fits are performed for each velocity. For each of the two

parameters an average value is calculated from these fits. These averages are used as starting values in an iterative procedure to obtain a global best fit, with $x_{\rightarrow}$ constraint to a single value (within 0.2 nm tolerance) and k_0 allowed to vary within a factor of 10.

The quality of the fits is characterised by the root mean squared error ($rmse$) :

$$rmse = \frac{1}{n_b}\sqrt{\sum_i (p_i - p_{hys,i})^2} \tag{7}$$

with p_i representing the experimental probability associated to the bin i, $p_{hys,i}$ the calculated probability for the bin i, and n_b the number of bins. For all histograms of this work, the $rmse$ are in the range of (1-5)$\times 10^{-3}$.

Influence of ΔG on the fitted parameters

At constant trap distance force flipping between the folded and unfolded states can be observed for hairpins DNA10, RNA10 and DNA18. We measure the force $F_{1/2}$, for which the average dwell times in the folded and unfolded states are equal. This allows us to experimentally estimate ΔG. We have $\Delta G = F_{1/2}L$, where L is the change in the hairpin length induced by a transition at the force $F_{1/2}$. Measured $F_{1/2}$ and derived ΔG values are presented in table S4. The table contains no line for RNA18, since flipping is not observed for this hairpin. The energies obtained from $F_{1/2}$ and the ΔG_{tot} values of Table 2 agree within 30%. Using the energies obtained from $F_{1/2}$ rather than the theoretically predicted ΔG_{tot} values in the fitting procedure changes neither $x_{\rightarrow}$ nor the ratios between the transition rates k_0, as can be seen by comparing Table S4 and Table 3.

S.2. Precision of the measured force hysteresis compared to the precision of separately measured folding and unfolding forces.

Calibration uncertainty limits the precision of the measured force to about 5 % in our experimental case. Major causes are variability in sample viscosity, uncertainty in the distance between the coverslip surface and the beads and variations in diameter, refractive index and deviations from spherical shape of the beads. These quantities influence the force calibration derived from the power spectral density of a captured bead. Experimental noise also affects the precision of the measured force and amounts to $\Delta F \simeq 0.25$ pN for the unfolding and folding forces and acquisition rates of this study. The precision is slightly lower for the determination of the zero-force baseline, possibly due to the close proximity of the beads that can lead to cross-talk and weak rearrangement of the beads in the traps. We estimate the precision of our zero-force baseline to $\Delta F_0 \simeq 0.5$ pN. Given these contributions, we can estimate the precision of the measured unfolding force ΔF_{un}, folding force ΔF_{fo} and force hysteresis ΔF_{hys} as follows.

$$\begin{aligned}\Delta F_{un} &= \Delta F + \Delta F_0 + 0.05(F_{un}) \\ &\simeq 0.75\text{pN} + 0.05(F_{un})\end{aligned}$$

$$\begin{aligned}\Delta F_{fo} &= \Delta F + \Delta F_0 + 0.05(F_{fo}) \\ &\simeq 0.75\text{pN} + 0.05(F_{fo})\end{aligned}$$

$$\begin{aligned}\Delta F_{hys} &= 2\,\Delta F + 0.05(F_{un} - F_{fo}) \\ &\simeq 0.5\text{pN} + 0.05(F_{un} - F_{fo})\end{aligned}$$

This simple analysis shows that we have a higher precision on the hysteresis measurement than on the opening and closing forces. Calculated ΔF_{un}, ΔF_{fo} and ΔF_{hys} for the four hairpins at 50 nm/s are provided in Table S1.

Table S1. Estimation of the error in the measured unfolding force, folding force and force hysteresis for the four hairpins at 50 nm/s.

hairpin	ΔF_{un} (pN)	ΔF_{fo} (pN)	ΔF_{hys} (pN)
DNA10	1.05	1.05	0.50
DNA18	1.05	0.90	0.65
RNA10	1.40	1.20	0.75
RNA18	1.50	1.05	0.90

S.3. Calculation of hysteresis histograms and associated error bars.

On a stretch and release cycle, the unfolding and the folding forces are the result of independent events, and are thus uncorrelated. Consequently, for a given molecule and at a given speed, the hysteresis $F_{un} - F_{fo}$ can be calculated combining values from different cycles. The histogram (for a given hairpin, at one speed) was obtained in three steps: first, all the unfolding forces measured were paired with all the folding forces measured on the same molecule in order to generate all possible hysteresis values. Second, the obtained values were weighted depending on the actual number of stretch and release cycles performed on this molecule. Third, a histogram was built from the values obtained with all the molecules probed. The number of event n_i in each histogram bin was normalised to the probability $p_i = n_i/N$, with N the total number of cycles performed. The error bars have been determined using the variance of the binomial law (10), leading to the following uncertainty in the probability: $p_i \pm (p_i(1 - p_i)/N)^{1/2}$. They are shown for every histogram of this publication.

S.4. Fit of the force-diplacement curves with the WLC model.

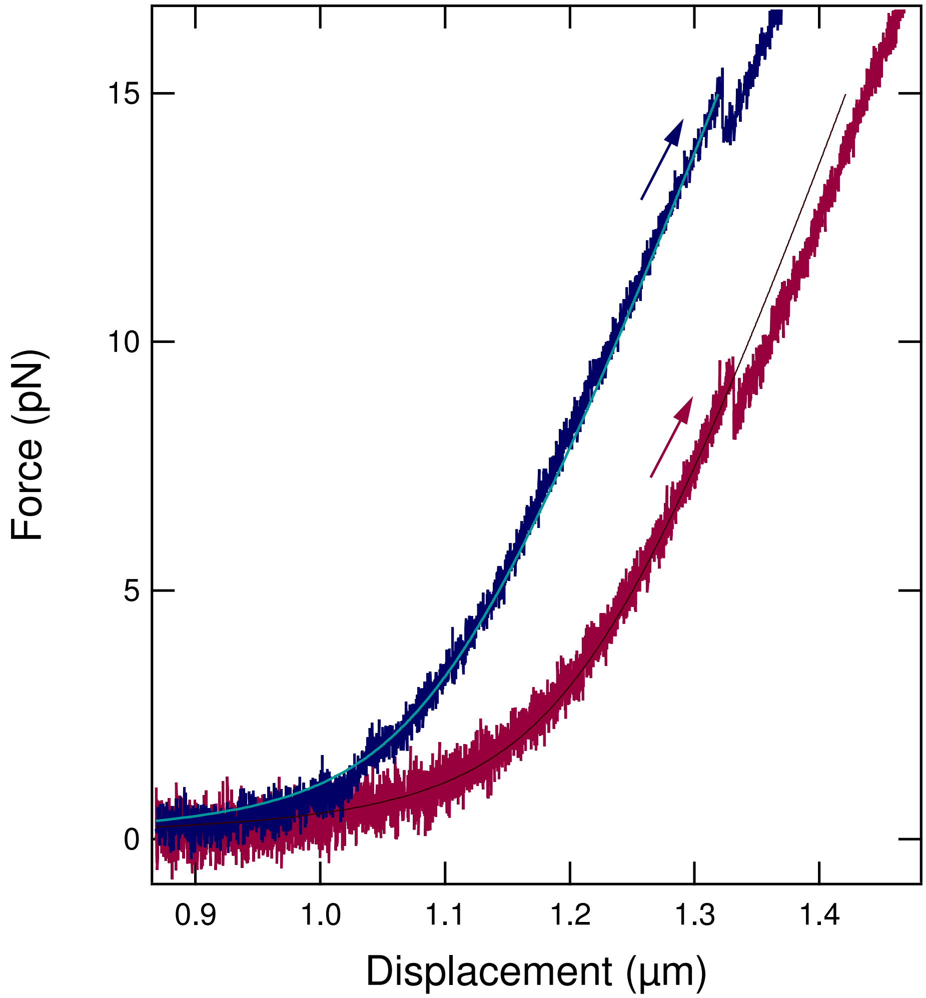

Figure S1. Force-displacement curves representing the stretching of RNA18 (blue) and DNA18 (red) at 50 nm/s, and fit to the WLC model. For DNA18, the obtained parameters are L_0=1.3 μm and L_p=43 nm respectively. These values are in good agreement with the expected ones for 3991 base pairs double stranded DNA molecules (11). For the DNA/RNA hybrid construct (RNA18), the crystallographic and persistance length are L_0=1.17 μm and L_p=47 nm respectively. Literature data are lacking for long DNA/RNA hybrid, but the obtained L_0 value quantitatively agree with the one expected for a double stranded RNA A-helix of the same number of base pairs (3991) (12).

S.5. Exemple of distributions of folding and unfolding forces.

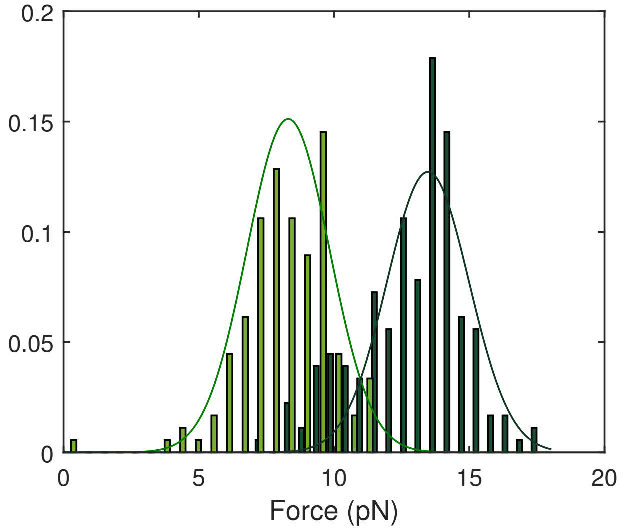

Figure S2. Distributions of unfolding (dark green) and folding (light green) forces for RNA10 at 50 nm/s. The mean opening force and the mean folding force are measured as the maximum of a gaussian fit to the corresponding distributions. The mean transition force F_t is the average of these two values.

S.6. Hysteresis distributions for the four hairpins at 50, 300, and 450 nm/s.

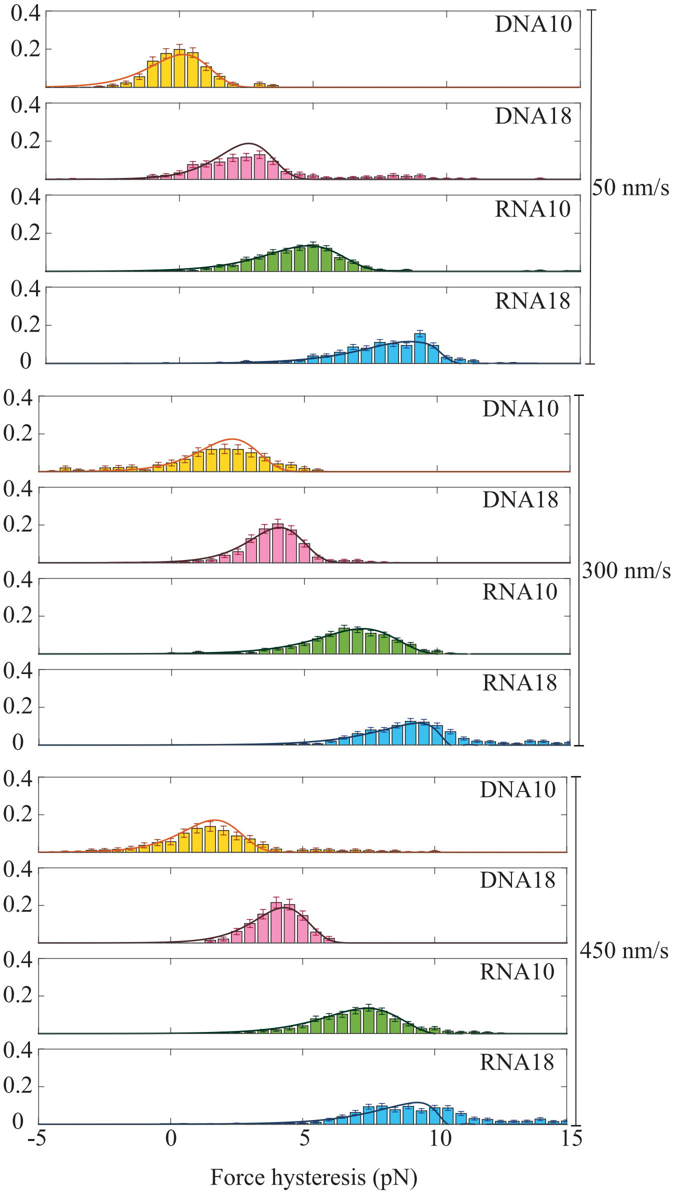

Figure S3. Histograms of the hysteresis measured on the four hairpins at 50 (top), 300 (middle), and 450 (bottom) nm/s. The solid lines correspond to the fit with the calculated convolutions of $p_{\rightarrow}$ and $p_{\leftarrow}$ (Eqs. 4-6). The number of measured molecules and cycles are given in Table S3. The *rmse* is in the range (1.0-5.0)$\times 10^{-3}$ for each fitted histogram.

S.7. Velocity dependance of the hysteresis distributions and number of measured molecules and cycles.

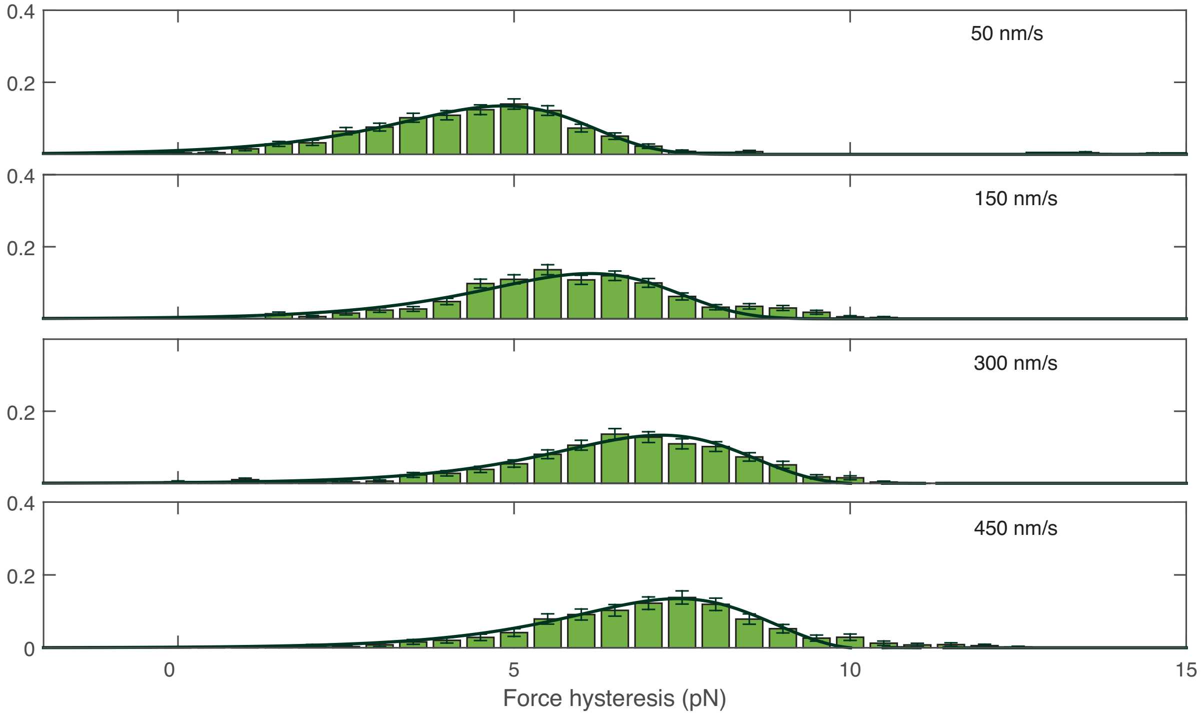

Figure S4. Histograms of the hysteresis measured on RNA10 at 50, 150, 300, and 450 nm/s. The solid lines correspond to the fit with the calculated convolutions of the unfolding and folding probabilities (Eqs. 4-6). Experimental data for 50 nm/s come from 148 stretch/release cycles on 48 molecules. 150 nm/s: 152 cycles on 44 molecules. 300 nm/s: 125 cycles on 16 molecules. 450 nm/s: 91 cycles on 7 molecules. There is good agreement between the experimental data and the calculated probability distributions. For this figure, the $rmse$ is in the range (1.4-2.7)$\times 10^{-3}$ for each panel.

S.8. Theoretical unfolding free-energy landscapes

In this section, we present energy landscapes obtained by sequential calculation of mfold free-energy contributions. We consider the expression

$$E_{tot}(j) = \sum_{i=1}^{j} E(i) - F\, l(j, F), \tag{8}$$

with

$$E(i) = \begin{cases} E_{bp}(i) + E_{stretch} & : \quad i \le n_{stem} \\ E_{loop}/n_{loop} + E_{stretch} & : \quad n_{stem} < i \le n_{stem} + n_{loop}, \end{cases}$$

where $n_{stem} = 13$, $n_{loop} = 5$ for DNA10 and RNA10 and $n_{loop} = 9$ for DNA18 and RNA18. The binding energies of the base pairs E_{bp} and the loop energies E_{loop} are obtained by mfold. $E_{stretch}$ denotes the energy required to extend a single-strand of two nucleotides from zero-force to F. The term $-F\ l(j, F)$ on the right of Eq.8 accounts for the mechanical work of the generated single-strand of j base pairs exhibiting a length $l(j, F)$ at force F. $E_{stretch}$ and $l(j, F)$ are determined as described in section S1. This one-dimensional theoretical description of the unfolding energy landscape has been used in earlier studies, for instance by the Block group (8) and our group (13, 14).

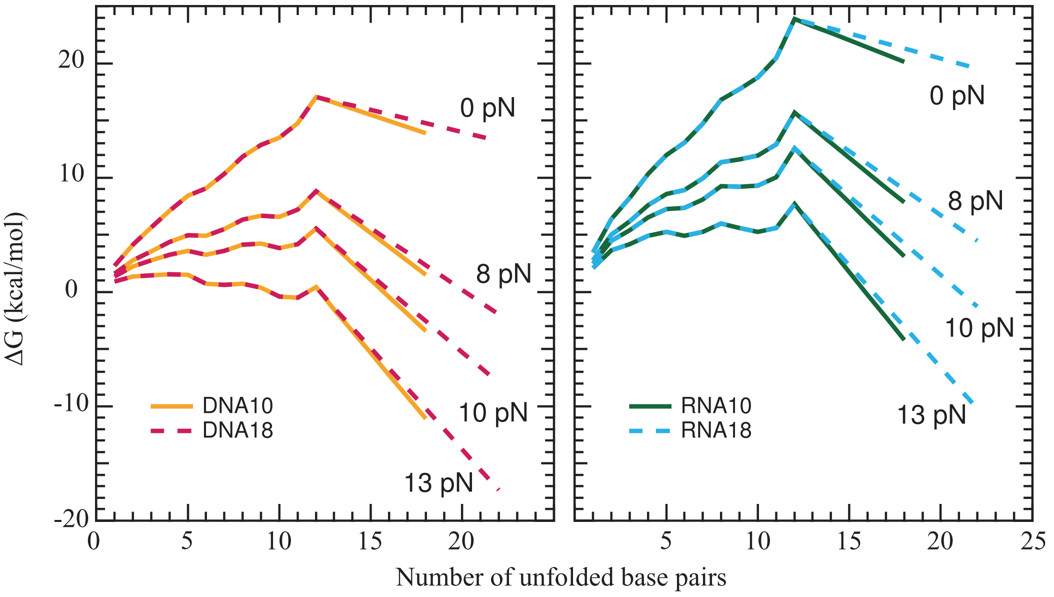

Figure S5. Unfolding energy landscapes calculated for the DNA (left panel) and RNA (right panel) hairpin structures at 0, 8, 10, and 13 pN externally imposed force. The dashed lines represent the large loop hairpins (DNA18 and RNA18), and the solid lines the smaller loop ones (DNA10 and RNA10). The stem of the two DNA (resp. RNA) hairpins being identical, at a given force the curves are superimposed from 1 to 12 unfolded base pairs.

In Fig. S5, we present the landscapes calculated for the hairpin

structures at different forces. In all cases of relevance to our measurements the maximum of the landscape occurs at the last base pair of the stem, i.e. at the top of the stem in the representation of main text Fig.1. The experimental results, however, indicate transition states that are closer to the bottom of the stem. A way to account for this discrepancy consists in assuming that the base pairs in the vicinity of the opening fork are destabilized. This is a way to introduce cooperativity. More pronounced destabilisation corresponds to stronger cooperativity and would lead to smaller $x_{\rightarrow}$.

As a main result this supplementary section indicates that cooperativity is required to account for the experimentally observed positions of the transition state. In agreement with the discussion section of the main text, our observation of significantly smaller $x_{\rightarrow}$ for RNA than for DNA suggests that cooperativity is stronger in RNA.

Table S2. Increase of hysteresis with displacement velocity. The values correspond to the maximum of the fit of the measured hysteresis distribution to our out-of-equilibrium model.

hairpin	hysteresis at 50 nm/s (pN)	hysteresis at 450 nm/s (pN)
DNA10	0	2
DNA18	2.5	4.5
RNA10	5	7.5
RNA18	8.5	9.5

Table S3. Number of molecules and stretch-release cycles that were measured and analyzed.

hairpin	50 nm/s (mol./cyc.)	150 nm/s (mol./cyc.)	300 nm/s (mol./cyc.)	450 nm/s (mol./cyc.)
DNA10	15 / 70	12 / 46	8 / 49	5 / 52
DNA18	15 / 70	13 / 95	7 / 68	6 / 51
RNA10	48 / 148	44 / 152	16 / 125	7 / 91
RNA18	29 / 114	21 / 150	17 / 122	9 / 124

Table S4. Force $F_{1/2}$ measured at constant trap distance, energy ΔG derived from $F_{1/2}$, and the parameters $x_{\rightarrow}$ and k_0 obtained by fitting our hysteresis measurements using ΔG derived from $F_{1/2}$.

hairpin	$F_{1/2}$ (pN)	ΔG (kJ/mol)	$x_{\rightarrow}$ (nm)	k_0 (s^{-1})
DNA10	6.3	56	4.4	$(2.4 - 7.1) \times 10^{-3}$
DNA18	7.0	70	4.6	$(1.0 - 2.5) \times 10^{-4}$
RNA10	11.0	117	3.1	$(1.8 - 3.0) \times 10^{-5}$

References

[1] Evans, E., and Ritchie, K. (1997) Dynamic strength of molecular adhesion bonds. *Biophys. J.*, **72**, 1541–1555.

[2] Kramers, H. A. (1940) Brownian motion in a field of force and the diffusion model of chemical reactions. *Physica*, **7**, 284–304.

[3] Bell, G. I. (1978) Models for the specific adhesion of cells to cells. *Science*, **200**, 618–627.

[4] Dudko, O. K., Hummer, G., and Szabo, A. (2006) Intrinsic rates and activation free energies from single-molecule pulling experiments. *PRL*, **96**, 108101.

[5] Pierse, C. A., and Dudko, O. K. (2013) Kinetics and energetics of biomolecular folding and binding. *Biophys. J.*, **105**, L19–L22.

[6] Bosco, A., Camunas-Soler, J., and Ritort, F. (2014) Elastic properties and secondary structure formation of single-stranded DNA at monovalent and divalent salt conditions. *Nucl. Acids Res.*, **42**, 2064–2074.

[7] Bizarro, C. V., Alemany, A., and Ritort, F. (2012) Non-specific binding of Na^{+} and Mg^{2+} to RNA determined by force spectroscopy methods. *Nucl. Acids Res.*, **40**, 6922–6935.

[8] Woodside, M. T., Behnke-Parks, W. M., Larizadeh, K. Travers, K., Herschlag, D., and Block, S. M. (2006) Nanomechanical measurement of the sequence dependent folding landscapes of single nucleic acid hairpins. *Proc. Natl. Acad. Sci.*, **103**, 6190–6195.

[9] McIntosh, D. B., Duggan, G., Gouil, Q., and Saleh, O. A. (2014) Sequence-dependent elasticity and electrostatics of single-stranded DNA: signatures of base-stacking. *Biophys. J.*, **106**, 659–666.

[10] van Kampen, N. G. (2007) *Stochastic Processes in Physics and Chemistry* Elsevier

[11] Wang, M. D., Yin, H., Landick, R., Gelles, J., and Block, S. M. (1997) Stretching DNA with Optical Tweezers. *Biophys. J.*, **72**, 1335–1346.

[12] Hagerman, P. J. (1997) Flexibility of RNA. *Annu. Rev. Biophys. Biomol. Struct.*, **126**, 139–156.

[13] Bockelmann, U., Thomen, P., Essevaz-Roulet, B., and Heslot, F. (2002) Unzipping DNA with optical tweezers: high sequence sensitivity and force flips. *Biophys. J.* **82**, 1537-1553.

[14] Bockelmann, U., and Viasnoff, V. (2008) Theoretical study of sequence-dependent nanopore unzipping of DNA. *Biophys. J.* **94**, 2716-2724.
